# Prognostic factors of noninvasive mechanical ventilation in lung cancer patients with acute respiratory failure

**DOI:** 10.1371/journal.pone.0191204

**Published:** 2018-01-12

**Authors:** Wei-Chih Chen, Vincent Yi-Fong Su, Wen-Kuang Yu, Yen-Wen Chen, Kuang-Yao Yang

**Affiliations:** 1 Department of Chest Medicine, Taipei Veterans General Hospital, Taipei, Taiwan; 2 School of Medicine, National Yang-Ming University, Taipei, Taiwan; 3 Department of Internal Medicine, Taipei City Hospital Yangming Branch, Taipei, Taiwan; 4 Institute of Emergency and Critical Care Medicine, School of Medicine, National Yang-Ming University, Taipei, Taiwan; Azienda Ospedaliero Universitaria Careggi, ITALY

## Abstract

**Introduction:**

Few studies have reported outcomes of lung cancer patients with acute respiratory failure (RF) using noninvasive positive pressure ventilation (NIPPV). The aim of this study was to investigate the prognostic factors in these patients.

**Materials and methods:**

This retrospective observational study included all hospitalized lung cancer patients who received NIPPV for acute RF. It was conducted at a tertiary medical center in Taiwan from 2005 to 2010. The primary outcome was all cause mortality at 28 days after the initiation of NIPPV. Secondary outcomes included all-cause in-hospital mortality, weaning from NIPPV, intubation rate, tracheostomy rate, duration of NIPPV, hospital stay and intensive care unit stay.

**Results:**

The all-cause mortality rate at day 28 of the enrolled 58 patients was 39.66%. The 90-day and 1-year mortality rates were 63.79% and 86.21%, respectively. NIPPV as the first line therapy for RF had higher 28-day mortality rate than it used for post-extubation RF (57.6% versus 16.0%, p<0.05). Independent predictors of mortality at 28 days were progressive disease or newly diagnosed lung cancer (OR 14.02 95% CI 1.03–191.59, p = 0.048), combined with other organ failure (OR 18.07 95% CI 1.87–172.7, p = 0.012), and NIPPV as the first line therapy for RF (OR 35.37 95% CI 3.30–378.68, p = 0.003).

**Conclusion:**

Lung cancer patients using NIPPV with progressive or newly diagnosed cancer disease, combined with other organ failure, or NIPPV as the first line therapy for respiratory failure have a poor outcome.

## Introduction

Lung cancer is the major cause of cancer-related death worldwide, reportedly accounting for 12.7% of new cancer cases and 18.2% of cases of cancer-associated mortality[1]. In addition, lung cancer-associated complications result in high rates of morbidity and mortality. A cohort study in two intensive care units (ICU) reported that the main reasons why lung cancer patients were admitted to the ICU included severe sepsis, septic shock, acute respiratory failure and cardiovascular complications, with mortality rates in the ICU and hospital of 42% and 59%, respectively[2]. Acute respiratory failure (RF) is a life-threatening complication of lung cancer patients and is usually associated with a poor prognosis[3]. In the recent Surveillance, Epidemiology, and End Results (SEER) Medicare registry study, 76% of the 49373 patients with lung cancer admitted to an ICU survived hospitalization. However, only 35% of these patients were still alive at 6 months after discharge. Use of mechanical ventilation was associated with an increased risk of mortality[4]. Besides, in the prospective multicenter Lung Cancer in Critical Care (LUCCA) Study, 53% patients required ventilatory support[5].

One of the most frequently encountered complications in critically ill cancer patients is RF[6]. In cancer patients with RF who require ventilator support, endotracheal intubation with invasive mechanical ventilation (IMV) is the most common treatment modality[7]. However, apart from patient discomfort during translaryngeal intubation, several adverse outcomes such as ventilator-associated pneumonia, barotrauma, and tracheal injury are often associated with the use of IMV[8]. To avoid these problems and increase patient comfort, noninvasive positive pressure ventilation (NIPPV) has been adopted in recent years. Previous studies have addressed the benefits of NIPPV in patients with hematologic malignancies and acute RF[9–12], however, few studies have investigated the use of NIPPV for lung cancer patients with acute RF. Meert et al reported that 21% of lung cancer patients in an ICU who used NIPPV as the initial support eventually required intubation, and their discharge rate from hospital was high at 47.4%[13]. In cancer patients with acute hypoxemic RF, NIPPV as first-line therapy with clinical failure had higher mortality compared with IMV[14]. Pulmonary infections and high severity scores had been associated with NIPPV failure[15]. NIPPV failure during acute respiratory distress syndrome in patients with cancer also has increased in-hospital mortality[16]. The aim of this study was to assess NIPPV in lung cancer patients with acute RF and to explore the prognostic factors for outcome analysis.

## Materials and methods

### Study design and patients

This retrospective study was conducted at Taipei Veterans General Hospital, a tertiary medical center in Taiwan. The investigation included all hospitalized lung cancer patients who received NIPPV for acute RF from January 2005 to September 2010. The diagnosis of lung cancer was established according to pathological evidence. Acute RF was classified into hypercapnia, hypoxemia, and mixed types. Patients using NIPPV after IMV to facilitate weaning from the post-operative care of lung cancer surgery were excluded. The study protocol was approved by the Institutional Review Board of Taipei Veterans General Hospital with a waiver of patient consent and was conducted in accordance with the Helsinki Declaration.

### Criteria of NIPPV

In this study, the initiation of NIPPV on acute respiratory failure included presence of one or more of following conditions: (1) severe dyspnea with active contraction of the accessory muscles or paradoxical abdominal movement, (2) tachypnea with rate more than 30 per minute, (3) poor oxygenation with ratio of partial pressure arterial oxygen and fraction of inspired oxygen less than 200 and respiratory acidosis with pH less than 7.35 and/or (4) partial pressure of carbon dioxide more than 45 mmHg. Instead, IMV was given if the patients have respiratory or cardia arrest, consciousness drowsy or under sedation, massive aspiration, inability to remove secretions, hemodynamic instability or life-threatening arrhythmia.

### Data collection and measurements

“Do-Not-Resuscitate” orders, Eastern Cooperative Oncology Group performance status[17], types and causes of acute RF were recorded in addition to demographic data, including age, gender, and comorbidities. Lung cancer-related information such as histology type, staging[18], previous anti-cancer treatment, recent therapy within 2 weeks before events of RF, and cancer status were also recorded. Respiratory and critical care data concerning the initial mode of mechanical ventilation, site of NIPPV use, oxygen therapy before NIPPV, arterial blood gas analysis at the onset of respiratory failure, serum albumin level, presence of organ failure, and severity score were also collected. The main outcome measure was all cause mortality at 28 days after the onset of RF. Secondary outcomes included all cause in-hospital mortality, intubation rate, tracheostomy rate, duration of noninvasive ventilation, hospital stay and ICU stay.

### Statistical analysis

The results were presented as mean ± standard deviation, median with interquartile range, or number (%) as appropriate. We used the Kolmogorov-Smirnov and Shapiro-Wilk tests to examine the normality of continuous variables. Independent *t* tests were used to compare normally distributed continuous variables, and the Mann-Whitney *U* test was used to compare non-normally distributed continuous variables. We used the Pearson *χ*^*2*^ test or the Fisher’s exact test to compare categorical variables. Variables showing with significant differences between groups were entered into univariate and multivariate logistic regression analyses using the enter method to determine factors independently associated with mortality. Odds ratios and 95% confidence intervals were also calculated. A *P* value less than 0.05 was considered to be statistically significant. All statistical analyses were performed using the Statistical Package for the Social Sciences (version 19.0, SPSS Inc., Chicago, IL).

## Results

### Patient characteristics and critical illness factors between survivors and non-survivors

**Fig 1:**
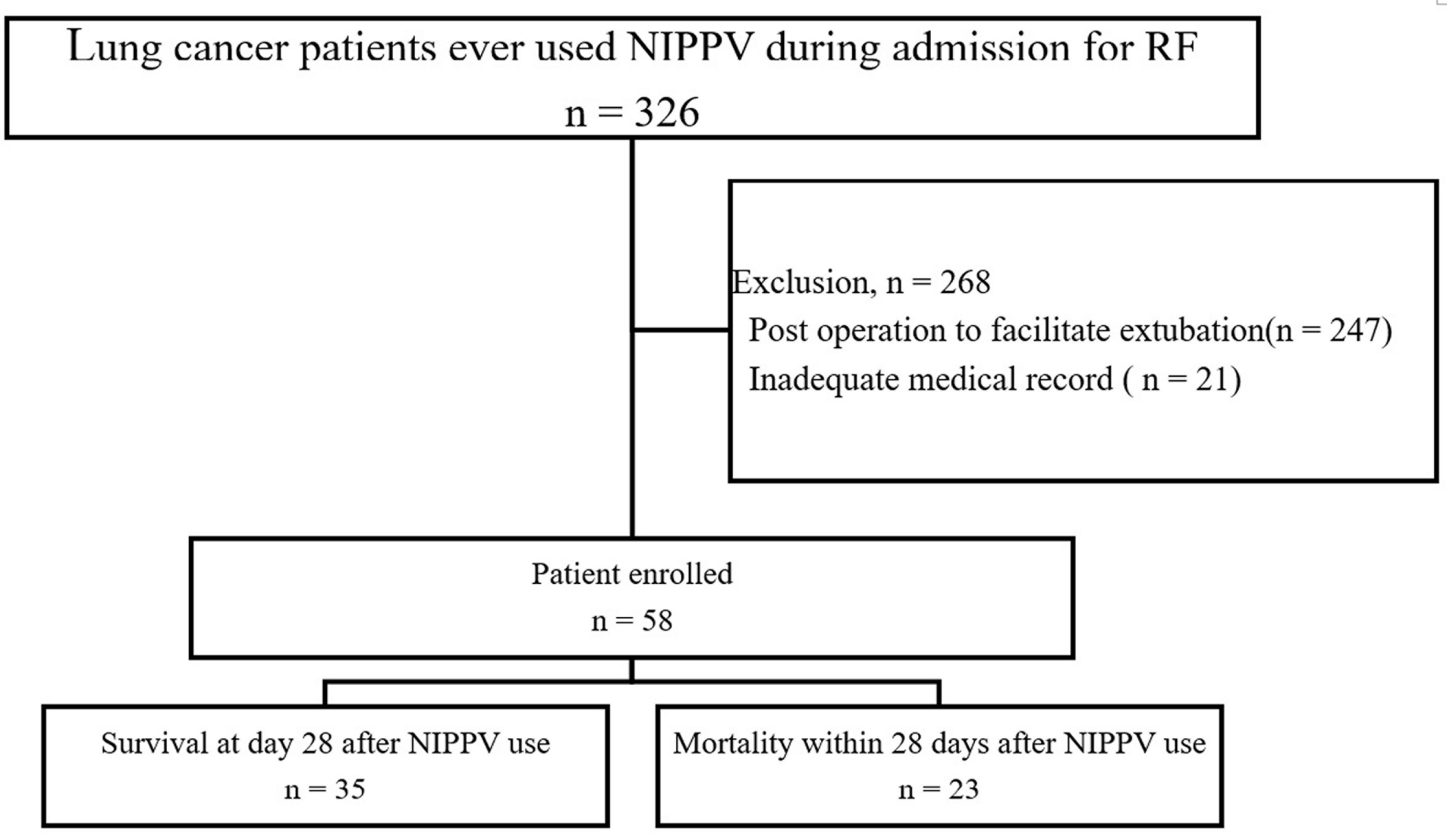
Flow chart of the study.

During the study period, 326 lung cancer patients received noninvasive ventilation therapy, of whom 247 patients were excluded owing to the use of noninvasive ventilation to facilitate extubation after thoracic surgery without evidence of post-extubation RF and 21 patients were excluded due to inadequate medical records, respectively. The remaining 58 patients were enrolled in the study, 35 of whom survived to day 28 (Fig 1). The characteristics of the patients are shown in Table 1. Respiratory status and cause of respiratory failure at NIPPV initiation are shown in S1 and S2 Tables. Survivors had significantly less advanced lung cancer and more stable disease or disease that was responsive to anti-cancer therapy. The critical illness-related factors are shown in Table 2. Survivors presented with more non-cancer-related RF and absence of other organ failure. In addition, NIPPV was used more often as the first line therapy for RF.

**Table 1:** Comparison of patient characteristics between survivors and non-survivors at day 28*.

Variables	Survivor (n = 35)	Non-survivor(n = 23)	P value
Age (years)	76.71 ± 9.49	75.97 ± 9.22	0.721
Gender (M/F)	25/10	18/5	0.561
Comorbidities			
DM	8 (22.9%)	4 (17.4%)	0.746
Hypertension	14 (40%)	14 (60.9%)	0.120
Chronic cardiovascular disease	3 (8.6%)	3 (13.0%)	0.673
Chronic respiratory disease	15 (42.9%)	9 (39.1%)	0.778
Chronic kidney disease	1 (2.9%)	4 (17.4%)	0.075
Chronic liver disease	1 (2.9%)	1 (4.3%)	1
Performance status			0.145
0~3	19 (54.3%)	8 (34.8%)	
4	16 (45.7%)	15 (65.2%)	
Cancer cell type			0.129
Small cell lung cancer	7 (20%)	1 (4.3%)	
Non-small cell lung cancer	28 (80%)	22 (95.7%)	
Stage			0.039
Stage I-III	17 (48.6%)	5 (21.7%)	
Stage IV	18 (51.4%)	18 (78.3%)	
Anti-cancer therapy^#^			0.872
Without anti-cancer therapy	10	8	
With surgery	2	1	
With target, radio-, or chemo-therapy only	23	14	
Tumor status			0.016
Stable disease or responsive to current therapy	13 (37.1%)	2 (8.7%)	
Progressive disease or newly diagnosed lung cancer	22 (62.9%)	21 (91.3%)	

Data are expressed as mean ± SD or No. (%). DM, diabetes mellitus.

*Survival was defined according to the survival status on day 28 of initiating noninvasive positive pressure ventilation.

^#^Before initiation of noninvasive positive pressure ventilation.

**Table 2:** Comparison of critical illness factors between survivors and non-survivors at day 28*.

Variables	Survivor (n = 35)	Non-survivor(n = 23)	P value
Types of RF			0.490
Hypoxemic	9 (25.7%)	3 (13%)	
Hypercapnic	12 (34.3%)	10 (43.5%)	
Mixed	14 (40%)	10 (43.5%)	
Causes of RF by cancer			<0.001
Cancer or treatment-related	14 (40.0%)	20 (87.0%)	
Non-cancer-related	21 (60.0%)	3 (13.0%)	
Severity score			
APACHEII	16.97 ± 5.41	20.36 ± 7.89	0.073
SAPSII	46.24 ± 11.90	48.67 ± 13.19	0.487
SOFA	4.91 ± 2.38	5.55 ± 3.36	0.414
Reasons for initiating NIPPV use for RF			0.001
Post-extubation RF	21 (60.0%)	4 (17.4%)	
As the first-line therapy	14 (40.0%)	19 (82.6%)	
Location where NIPPV was performed			0.460
General ward	5 (14.3%)	3 (13.0%)	
ICU	30 (85.7%)	19 (82.6%)	
Hospice ward	0	1 (4.3%)	
Serum albumin levels before NIPPV use (g/dl)	2.63 ± 0.64	2.64 ± 0.61	0.943
Other organ failure			
Shock	4 (11.4%)	7 (30.4%)	0.093
Renal	10 (28.6%)	10 (43.5%)	0.243
Liver	4 (11.4%)	3 (13.0%)	1
Metabolic acidosis	8 (22.9%)	4 (17.4%)	0.746
Coagulopathy	6 (17.1%)	4 (17.4%)	1
Numbers of organ failure			0.034
RF only	19 (54.3%)	6 (26.1%)	
Combined other organ failures	16 (45.7%)	17 (73.9%)	

Data are expressed as mean ± SD or No. (%).APACHEII, Acute Physiology and Chronic Health Evaluation II score; ICU, intensive care unit; IMV, invasive mechanical ventilation; NIPPV, noninvasive positive pressure ventilation; RF, respiratory failure; SAPSII, Simplified Acute Physiology II Score; SOFA, Sequential Organ Failure Assessment.

*Survival was defined according to the survival status on day 28 of initiating NIPPV.

### Outcomes of the enrolled patients

The all-cause mortality rate at day 28 after the initiation of NIPPV was 39.66%, and the 90-day and 1-year mortality rates were 63.79% and 86.21%, respectively. Outcomes of the enrolled patients are presented in Table 3. Survivors had a significantly longer hospital stay than non-survivors; however, there were no significant differences in intubation rate, tracheostomy rate, duration of NIPPV, or ICU stay.

**Table 3:** Comparison of patient outcomes between survivors and non-survivors at day 28*.

Variables	Survivors (n = 35)	Non-survivors (n = 23)	P value
Hospital mortality	8 (22.9%)	23 (100%)	<0.001
Intubation after NIPPV	7 (20.0%)	5 (21.7%)	1
Tracheostomy after NIPPV	1 (2.9%)	0	1
Duration of NIPPV (days)	11.23 ± 16.98	6.35 ± 5.60	0.702
Hospital stay (days)	41.09 ± 26.37	18.91 ± 10.13	<0.001
ICU stay (days)	18.34 ± 17.78	12.40 ± 7.11	0.228

Data are expressed as mean ± SD or No. (%). ICU, intensive care unit; NIPPV, noninvasive positive pressure ventilation.

*Survival was defined according to the survival status on day 28 after initiating NIPPV.

To further elucidate clinical predictors of mortality among lung cancer patients with NIPPV, we used univariate and multivariate logistic regression analyses (Table 4). Significant variables including terminal stage, progressive disease or newly diagnosed lung cancer, cancer- or treatment-related respiratory failure, NIPPV as first line therapy for RF, and combined with other organ failure. Progressive disease or newly diagnosed lung cancer (OR 14.02, CI [1.03–191.59], p = 0.048), NIPPV as the first line therapy for RF (OR 35.37, CI [3.30–378.68], p = 0.003) and combined with other organ failure (OR 18.07, CI [1.89–172.7], p = 0.012) remained independent predictors after multivariate regression analysis.

**Table 4:** Predictors of mortality at day 28 after the onset of respiratory failure using univariate and multivariate logistic regression analyses.

Variables	Univariate			Multivariate		
	Odds ratio	95% confidence interval	P value	Odds ratio	95% confidence interval	P value
Terminal stage (stage IV)	3.40	1.03–11.20	0.044			
Progressive disease or newly diagnosed lung cancer	6.21	1.25–30.87	0.026	14.02	1.03–191.59	0.048
Cancer or treatment-related RF	10.00	2.49–40.12	0.001			
NIPPV as the first line therapy for RF	7.125	1.995–25.441	0.002	35.37	3.30–378.68	0.003
Combined other organ failures	3.37	1.07–10.56	0.038	18.07	1.89–172.70	0.012

IMV, invasive mechanical ventilation; NIPPV, noninvasive positive pressure ventilation; RF, respiratory failure.

**Fig 2:**
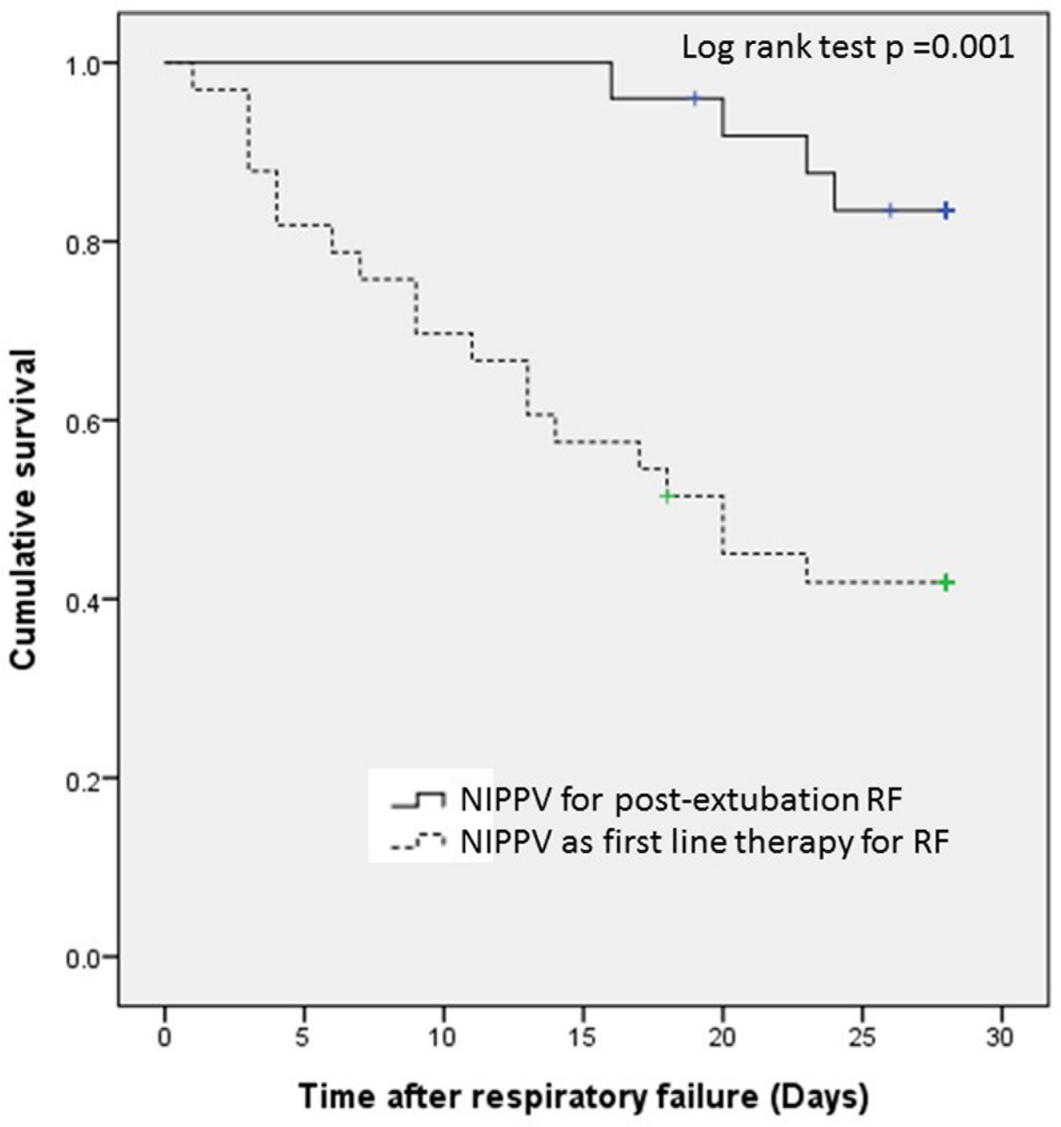
Patients’ survival according to reasons for NIPPV use in respiratory failure.

Kaplan-Meier analysis (Fig 2) shows poor survival among the patients initiating NIPPV as first line therapy for RF (log-rank test p = 0.001). Poorer survival in the patients with progressive disease or newly diagnosed lung cancer (log-rank test p = 0.033) is revealed in S1 Fig. The overall 1-year survival rate of the lung cancer patients with NIPPV was only 13.79% (S2 Fig).

## Discussion

In this study, the 28-day mortality and all-cause in-hospital mortality rates were high in adult lung cancer patients who received non-invasive ventilation for acute respiratory failure. The independent predictors for 28-day mortality included progressive disease or newly diagnosed lung cancer, NIPPV as first line therapy for respiratory failure and combined with other organ failure. However, comorbidities, performance status, cancer cell type, severity score, and serum albumin levels were not related to the survival of NIPPV therapy.

Due to different cultural backgrounds and policies of insurance reimbursement, some patients who receive noninvasive ventilation remained in the general ward or hospice ward instead of an ICU in Taiwan. However, the all-cause mortality rates in this study were similar to those in other studies on critically ill lung cancer patients[2,4,13,19–21], ranging from 54% to 59%. Consistent with these studies, mortality was not influenced by where noninvasive ventilation was performed. The 6-month survival rate of lung cancer patients admitted to an ICU has been reported to be 27%[19] to 35%[4] in various studies. In the current study, the 1-year mortality rate of the patients who received noninvasive ventilation was as high as 86.2%, indicating the extremely poor prognosis of such patients.

Both patient characteristics and critical illness-related factors have been reported to have an impact on mortality. In studies on lung cancer patients admitted to an ICU, severe comorbidities[2], poor performance status[14.22], cancer with disease progression[2,14,22], airway obstruction due to cancer,^2^ increased organ failure[2,21,22], thrombocytopenia[21], use of vasopressors[20,21], use of mechanical ventilation[4,19,21], and acute respiratory failure[19] have been associated with increased ICU, hospital and 6-month mortality rates. We also found that NIPPV as first line therapy for RF was associated with an increased 28-day mortality rate. The exact reason for this is unknown; however a possible explanation is that most of our patients suffered from non-cancer-related acute RF with other organ failure and IMV has been reported to be more suitable for these patients[23].

Noninvasive ventilation is also an effective method to facilitate weaning from IMV. In this study, we observed that most patients who received IMV had RF due to non-cancer-related causes, which are often reversible if aggressive treatment is provided. Transitioning from invasive to noninvasive ventilation in such patients is usually performed when their general condition improves. In addition, noninvasive ventilation can prevent many of the complications associated with IMV such as ventilator-associated pneumonia, barotrauma or airway injury. Several clinical trials and meta-analyses have favored the use of noninvasive ventilation after extubation to prevent post-extubation respiratory failure[24–28]. Among these studies, patients with chronic respiratory disorders, especially chronic obstructive pulmonary disease, were found to benefit the most. Due to the high prevalence of chronic respiratory diseases in our patients, using noninvasive ventilation to facilitate the early removal of endotracheal tubes was a reasonable and acceptable therapeutic modality.

There are several limitations to this study. First, our cohort was derived from a single tertiary teaching medical center in Taiwan. Secondly, the relatively small sample size and exclusion of some patients due to inadequate medical records might have biased the results. Third, some patients had “Do-Not-Resuscitate” orders in our study. However, most of our patients signed the non-resuscitation permits after initial stabilization of acute episode, the medical decisions to choose the initial types of mechanical ventilation were not influenced by the DNR order. Finally, because this is a retrospective study, some confounding factors may have been neglected.

In conclusion, progressive disease or newly diagnosed lung cancer and multiple organ failure have poor prognosis. In addition, NIPPV as first line therapy for RF is the most important predictor for 28-day mortality. The relative risk should be considered before initiation of NIPPV in lung cancer patients with acute RF.

## Supporting information

S1 Fig NIPPV Patients’ survival according to tumor status. NIPPV, non-invasive positive pressure ventilation.
(DOC)

S2 Fig Overall survival after 1-year follow up. (DOCX)

S1 Table Respiratory status at NIPPV initiation. NIPPV, non-invasive positive pressure ventilation.
(DOC)

S2 Table Causes of respiratory failure. (DOCX)

S3 Table Predictors of mortality at day 28 after the onset of respiratory failure using univariate and multivariate logistic regression analyses with forward likelihood ratio method. (DOCX)

S4 Table NIPPV setting and complications. (DOC)
