# Supplementary figures and images for: Prognostic factors of noninvasive mechanical ventilation in lung cancer patients with acute respiratory failure
Source: PLoS One. 2018 Jan 12;13(1):e0191204. doi: 10.1371/journal.pone.0191204 (PMC5766147; doi:10.1371/journal.pone.0191204)

**S1 Fig. NIPPV Patients’ survival according to tumor status.**


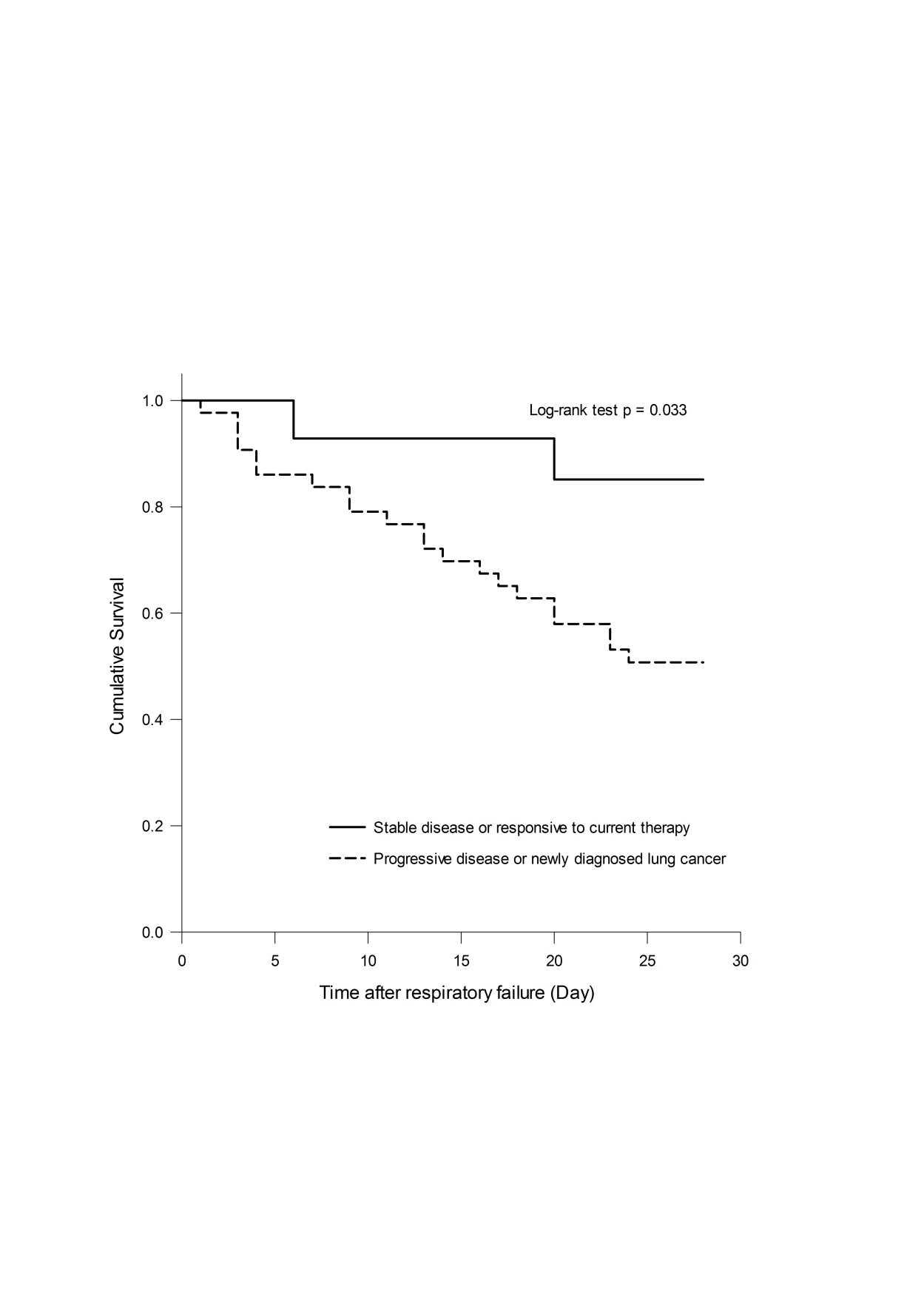

Supplement: S1 Fig — NIPPV, non-invasive positive pressure ventilation. (DOC) [file pone.0191204.s001.doc]

**S2 Fig. Overall survival after 1-year follow up.**


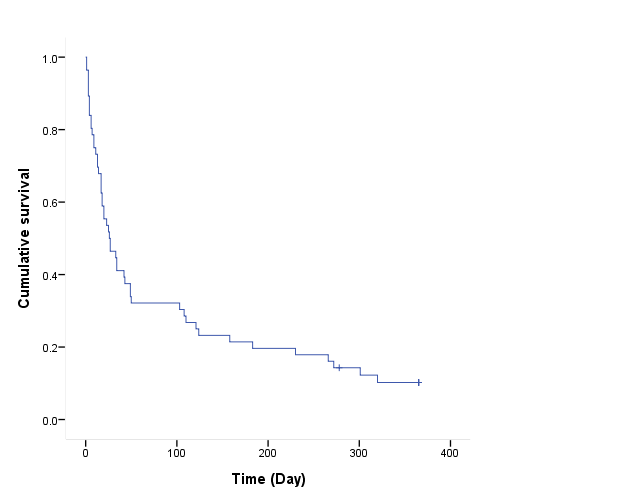

Supplement: S2 Fig — (DOCX) [file pone.0191204.s002.docx]
