# Supplementary material for: Prognostic factors of noninvasive mechanical ventilation in lung cancer patients with acute respiratory failure
Source: PLoS One. 2018 Jan 12;13(1):e0191204. doi: 10.1371/journal.pone.0191204 (PMC5766147; doi:10.1371/journal.pone.0191204)
Supplement: S1 Table — NIPPV, non-invasive positive pressure ventilation. (DOC) [file pone.0191204.s003.doc]

**S1 Table. Respiratory status at NIPPV initiation.**

| Variables | Survivor  (n=35) | Non-survivor (n=23) | P value |
| --- | --- | --- | --- |
| pH | 7.34 ± 0.12 | 7.34 ± 0.14 | 0.989 |
| PaO_2_ | 139.97 ± 105.04 | 110.47 ± 94.07 | 0.281 |
| PaCO_2_ | 50.42 ± 25.41 | 63.23 ± 28.23 | 0.078 |
| HCO_3_ | 25.32 ± 8.77 | 31.57 ± 11.12 | 0.020 |

HCO_3_, bicarbonate; NIPPV, noninvasive positive pressure ventilation; PaO_2_, arterial partial pressure of oxygen; PaCO_2_, arterial partial pressure of carbon dioxide.
