# Supplementary material for: Prognostic factors of noninvasive mechanical ventilation in lung cancer patients with acute respiratory failure
Source: PLoS One. 2018 Jan 12;13(1):e0191204. doi: 10.1371/journal.pone.0191204 (PMC5766147; doi:10.1371/journal.pone.0191204)
Supplement: S2 Table — (DOCX) [file pone.0191204.s004.docx]

**S2 Table. Causes of respiratory failure.**

| **Variables** | **Survivor**  **(n=35)** | **Non-survivor (n=23)** | **P value** |
| --- | --- | --- | --- |
|  |  |  | 0.001 |
| Cancer progression | 10 (28.6%) | 19 (82.6%) |  |
| Cancer treatment related | 4 (11.4%) | 1 (4.3%) |  |
| Sepsis | 8 (22.9%) | 0 |  |
| COPDAE | 10 (28.6%) | 0 |  |
| Others | 3 (8.5%) | 3 (13.1%) |  |

COPDAE, chronic obstructive pulmonary disease with acute exacerbation.
