# Supplementary material for: Prognostic factors of noninvasive mechanical ventilation in lung cancer patients with acute respiratory failure
Source: PLoS One. 2018 Jan 12;13(1):e0191204. doi: 10.1371/journal.pone.0191204 (PMC5766147; doi:10.1371/journal.pone.0191204)
Supplement: S3 Table — (DOCX) [file pone.0191204.s005.docx]

**S3 Table. Predictors of mortality at day 28 after the onset of respiratory failure using univariate and multivariate logistic regression analyses with forward likelihood ratio method.**

| Variables | Univariate | | | Multivariate | | |
| --- | --- | --- | --- | --- | --- | --- |
|  | Odds ratio | 95% confidence interval | P value | Odds ratio | 95% confidence interval | P value |
| Progressive disease or newly diagnosed lung cancer | 6.21 | 1.25-30.87 | 0.026 | 12.32 | 1.02-149.18 | 0.048 |
| Cancer or treatment-related RF | 10.00 | 2.49-40.12 | 0.001 |  |  |  |
| NIPPV as the first line therapy for RF | 7.125 | 1.995-25.441 | 0.002 | 34.08 | 3.24-358.73 | 0.003 |
| Combined other organ failures | 3.37 | 1.07-10.56 | 0.038 | 17.73 | 1.86-169.33 | 0.013 |

IMV, invasive mechanical ventilation; NIPPV, noninvasive positive pressure ventilation; RF, respiratory failure.
