# Supplementary material for: Prognostic factors of noninvasive mechanical ventilation in lung cancer patients with acute respiratory failure
Source: PLoS One. 2018 Jan 12;13(1):e0191204. doi: 10.1371/journal.pone.0191204 (PMC5766147; doi:10.1371/journal.pone.0191204)
Supplement: S4 Table — (DOC) [file pone.0191204.s006.doc]

**S4 Table. NIPPV setting and complications**

| Variables | Results |
| --- | --- |
| Models of NIPPV | VPAP (ResMed, Bella Vista, Australia) |
| Modes | Pressure support ventilation |
| Interface | Oronasal mask |
| Initial setting | IPAP 20 cmH_2_O, EPAP 5cmH_2_O, backup rate 15/min |
| Complications |  |
| Mask discomfort | 25 (43.1%) |
| Skin abrasion | 12 (20.7%) |
| Abdominal distention | 10 (17.2%) |

EPAP, expiratory positive airway pressure; IPAP, inspiratory positive airway pressure; NIPPV, noninvasive positive pressure ventilation.
